# Hypothalamic injury in spontaneous subarachnoid hemorrhage: a diffusion tensor imaging study

**DOI:** 10.1007/s10286-020-00747-5

**Published:** 2020-11-28

**Authors:** Sung Jun Lee, Sung Ho Jang

**Affiliations:** Department of Physical Medicine and Rehabilitation, College of Medicine, Yeungnam University, 317-1, Daemyungdong, Namku, Taegu, 705-717 Republic of Korea

**Keywords:** Hypothalamus, Subarachnoid hemorrhage, Diffusion tensor imaging, Fractional anisotropy, Apparent diffusion coefficient

Dear Editors,

Spontaneous subarachnoid hemorrhage (SAH), which accounts for 3% to 5% of all strokes, is mainly the result of the extravasation of blood into the subarachnoid space, caused by aneurysmal rupture of the cerebral artery [1]. It has been reported that more than half of patients with SAH show neurologic manifestations related to brain injury [1]. Several pathophysiologic mechanisms involved in SAH-related brain injury have been suggested, including global vasogenic edema, vasospasm and cerebral ischemia, mechanical injury (via increased intracranial pressure (barotrauma from pressure waves) or a direct mass effect of the SAH), and chemical injury (a blood clot can cause neural injury through the release of potentially damaging substances, such as free iron, which may result in the generation of free radicals or inflammatory cytokines) [2]. Through these SAH-related mechanisms, a SAH can affect extensive brain areas, including specific neural structures such as the cingulum, fornix, and mamillothalamic tract [3].

Various clinical manifestations related to autonomic dysfunction in SAH have been reported, including hyperthermia, hypertension, cardiac dysrhythmia, impaired consciousness, and hyperhidrosis [4]. Among the sequelae of SAH, paroxysmal sympathetic hyperactivity, which is caused by dysfunction of portions of the central autonomic network (including the hypothalamus, amygdala, insular cortex, and brainstem), is a representative SAH-related autonomic disease [5]. The incidence of paroxysmal sympathetic hyperactivity of patients with SAH has been reported to be approximately 11.3%.

The hypothalamus, a central mediator of the autonomic nervous system, has essential roles in the autonomic functions of the human body (e.g., thermoregulation, regulation of food intake and body water content, endocrine control, reproduction, and circadian rhythm) [6]. Clinical manifestations caused by autonomic system dysfunction due to the hypothalamic injury are disturbance of thermoregulation, impaired consciousness, and hyperhidrosis, and these manifestations overlap with those of SAH [6]. Because the hypothalamus is anatomically located adjacent to the subarachnoid space and large subarachnoid cisterns, including the interpeduncular and chiasmatic cisterns, the hypothalamus could be vulnerable to SAH (supplemental fig. 1-A). However, precise estimation of the hypothalamus in the live human brain has been limited because of the anatomical characteristics of the hypothalamus, which is very small and only occupies approximately 0.5% of the whole brain volume; moreover, it has a deep location within brain white matter [7]. Regardless, diffusion tensor imaging (DTI) has enabled evaluation of the hypothalamus in the live human brain. Several studies using DTI have reported on hypothalamic injuries in patients with various brain diseases, including traumatic brain injury, hypoxic-ischemic brain injury, and multiple sclerosis [8–10]. However, no DTI study on the hypothalamic injury in patients with SAH has been reported.

In this study, we hypothesized that the hypothalamic injury could occur in the patients with SAH and investigated it using DTI. Detailed methods, demographic data, and clinical information are provided in supplemental file 1.

The results of the comparison of the DTI parameters of the patient and control groups are summarized in Table 1. The mean fractional anisotropy (FA) value was significantly lower in the patient group than in the control group (*p* < 0.05), whereas the mean apparent diffusion coefficient (ADC) value was significantly higher in the patient group than in the control group (*p* < 0.05).

**Table 1:** Comparison of the diffusion tensor imaging parameters between the patient and control groups

	FA	ADC
Patient group	0.23 ± 0.03	1.54 ± 0.23
Control group	0.26 ± 0.03	1.02 ± 0.13
*p* value	0.001*	0.001*

Values represent mean (± standard deviation)

*FA* fractional anisotropy, *ADC* apparent diffusion coefficient

*Significant differences between the patient and control groups, *p* < 0.05

The results of correlations between clinical information and the DTI parameters are provided in supplemental file 1.

In the current study, by using DTI, the hypothalamus was estimated in control subjects and patients with spontaneous SAH. The FA value was lower, and the ADC value higher, in the patient group than in the control group. Regarding DTI parameters, FA, which is a scalar measure, represents the level of white matter organization and is influenced by axonal myelination, density, and the degree of directionality and level of organization of white matter tracks. A decrement in the FA value indicates impaired integrity of the white matter. The ADC value is indicative of the magnitude of water diffusion in brain tissue, which can increase with some disease forms, particularly vasogenic edema, or through the accumulation of cellular debris from neuronal injury. Therefore, the statistically significant decrement in FA value and increment in ADC value appear to indicate the presence of injuries of the hypothalamus in the SAH patients. We think that the hypothalamic injury might be related with the blood in the subarachnoid space and large subarachnoid cisterns including the interpeduncular and chiasmatic cisterns adjacent to the hypothalamus. However, we were unable to discern whether the injuries were associated with mechanical or chemical factors. Therefore, further studies on this topic are warranted. Additional discussion is provided in supplemental file 1.

In conclusion, by using DTI, we demonstrated hypothalamic injuries in patients with spontaneous SAH. The results of this study suggest that our methodology could be helpful in research on the hypothalamus of patients with SAH. Accurate DTI-based estimation of the hypothalamus is deemed necessary in SAH patients showing clinical manifestations of autonomic dysfunction.

## Electronic supplementary material

Below is the link to the electronic supplementary material.

Supplementary file1 (PDF 894 kb)

Supplementary file2 (TIF 1,594 kb)
