# Supplementary material for: Hypothalamic injury in spontaneous subarachnoid hemorrhage: a diffusion tensor imaging study
Source: Clin Auton Res. 2020 Nov 28;31(2):321–2. doi: 10.1007/s10286-020-00747-5 (PMC8041696; doi:10.1007/s10286-020-00747-5)
Supplement: Supplementary file 1 — Supplementary file1 (PDF 894 kb) [file 10286_2020_747_MOESM1_ESM.pdf]

Supplemental Methods

Subjects

Seventeen patients (6 men, 11 women; mean age 57.65 ± 8.29 years; range, 45–69 years) with spontaneous SAH and 18 age- and sex-matched healthy control subjects (7 men, 11 women; mean age 51.22 ± 11.73 years; range, 38–77 years) with no history of neurological/psychiatric disease or head trauma were recruited to the study. Inclusion criteria for the 17 patients were as follows: (1) first-ever stroke; (2) spontaneous SAH due to aneurysmal rupture and confirmed by a neuroradiologist; and (3) DTI data obtained during the chronic SAH stage (i.e., >4 weeks after onset). Patients with intracerebral hemorrhage, intraventricular hemorrhage, hydrocephalus, or a definite lesion in the brain parenchyma were excluded. No significant difference in age or sex distribution was observed between the patient and control groups ($p > 0.05$). This study was performed retrospectively, and the study protocol was approved by the institutional review board of our university hospital.

Diffusion tensor imaging

DTI data were acquired at an average of 1.47 ± 1.01 months after the onset of SAH by using a 1.5 T Philips Gyroscan Intera scanner (Philips, Best, Netherlands) equipped with a Synergy-L sensitivity encoding (SENSE) head coil in order to obtain single-shot, spin-echo, planar-imaging pulse sequences with 32 non-collinear diffusion-sensitizing gradients. For each of the 32 non-collinear diffusion-sensitizing gradients, 67 contiguous slices were acquired parallel to the anterior commissure–posterior commissure line. Imaging parameters were as follows: acquisition matrix = 96 × 96, reconstructed to matrix = 192 × 192 matrix, field of view = 240 mm × 240 mm, TR = 10,398 ms, TE = 72 ms, parallel imaging reduction

factor (SENSE factor) = 2, EPI factor = 59 and b = 1000 s/mm^2, NEX= 1, slice gap = 0, and slice thickness = 2.5 mm. Analysis of the diffusion-weighted imaging data was performed by using tools within the Oxford Centre for Functional Magnetic Resonance Imaging of the Brain (FMRIB) Software Library (www.fmrib.ox.ac.uk/fsl). Eddy current-induced image distortions were removed using affine multi-scale two-dimensional registration as provided by the Oxford Centre for Functional Magnetic Resonance Imaging of Brain Software Library.. DTI-Studio software (CMRM, Johns Hopkins Medical Institute, Baltimore, MD) was used for evaluation of the hypothalamus. The region of interest (ROI) of the hypothalamus was identified by its location near the optic tract as the anterior boundary and mammillary body as the posterior boundary at the level of the upper midbrain. In DTI-studio, we used a ROI tool of rectangular shape to draw the ROI of the hypothalamus. The fractional anisotropy (FA) and apparent diffusion coefficient (ADC) values were obtained using a ROI of the hypothalamus (supplemental fig. 1-B) [1].

Figure 1

Statistical analysis

Statistical analysis was conducted by using SPSS 21.0 for Windows (SPSS, Chicago, IL, USA). The chi-squared test was performed to evaluate differences in the sex compositions of the patient and control groups. The independent t-tests were undertaken to assess differences in ages and DTI parameters between the patient and control groups. Statistical significance was accepted for p values < 0.05. The Spearman correlation test was used to determine the level of correlation between clinical information and the DTI parameters. A correlation

coefficient > 0.60 indicated a strong correlation, a correlation coefficient between 0.40 and 0.59 indicated moderate correlation, that between 0.20 and 0.39 indicated a weak correlation, and a correlation coefficient of ≤ 0.19 indicated a very weak correlation [2].

Supplemental Results

The correlations between clinical informations and DTI parameters are summarized in the supplemental table 3. There was no significant correlation between clinical informations and DTI parameters.

Supplemental Discussion

Since the introduction of DTI, a few studies have reported on hypothalamic injuries associated with various brain pathologies [3-6]. In 2012, Menzler et al., using tract-based spatial statistics, reported decrements in FA values in various brain areas, including the hypothalamus, and they observed a positive correlation with hypersomnia in patients with idiopathic narcolepsy [3]. Subsequently, Shen et al. [2014] demonstrated decrements of FA values in extensive brain areas, including the hypothalamus, and observed a positive correlation with the severity of depression in patients with multiple sclerosis [4]. In 2016, Jang et al. reported that hypothalamic injury (lower FA and higher ADC values) is related to a subjective indicator of excessive daytime sleepiness in patients with mild traumatic brain injury [5]. In 2018, Jang and Kwon, by using DTI, demonstrated that the decrement in FA and increment in ADC of the hypothalamus were related to dysautonomia in a patient with hypoxic-ischemic brain injury [6]. As a result, to the best of our knowledge, this is the first study to demonstrate the presence of hypothalamic injury in patients with spontaneous SAH.

However, some limitations of this study should be mentioned: (1) a small number of patients were recruited to this study; (2) definition of the ROI for measurement of DTI parameters is operator dependent and identification of the hypothalamus, which is a relatively small structure, is difficult due to low imaging resolution; (3) DTI parameters on the hypothalamus could be affected by the partial volume effect, which indicates a loss of contrast between adjacent tissues, such as between hypothalamic tissue and the cerebrospinal fluid, when two or more compartments are within a single imaging voxel; and (4) because this study was conducted retrospectively, we could not provide detailed clinical data that may have been related to the hypothalamic injury and correlate the clinical data and values of FA

and ADC of the hypothalamus. Therefore, we suggest that further prospective studies, including large numbers of patients, should be encouraged.

Supplemental References

1. Duvernoy HM, Bourgouin P (1999) The human brain: surface, three-dimensional sectional anatomy with MRI, and blood supply, 2nd completely rev. and enl. ed. Wien, New York, Springer.
2. Cohen J (1988) Statistical power analysis for the behavioral sciences, ed 2nd, Hillsdale, N.J., L. Erlbaum Associates.
3. Menzler K, Belke M, Unger MM, Ohletz T, Keil B, Heverhagen JT et al (2012) DTI reveals hypothalamic and brainstem white matter lesions in patients with idiopathic narcolepsy. Sleep Med 13:736–742
4. Shen Y, Bai L, Gao Y, Cui F, Tan Z, Tao Y et al (2014) Depressive symptoms in multiple sclerosis from an in vivo study with TBSS. Biomed Res Int 2014:148465.
5. Jang SH, Yi JH, Kim SH, Kwon HG (2016) Relation between injury of the hypothalamus and subjective excessive daytime sleepiness in patients with mild traumatic brain injury. J Neurol Neurosurg Psychiatry 87:1260–1261.
6. Jang SH, Kwon HG (2018) Injury of the hypothalamus in patients with hypoxic-ischemic brain injury. Am J Phys Med Rehabil 97:160-163.

Supplemental figure legend

Fig. 1. (A) The anatomy of the subarachnoid cisterns (chiasmatic and interpeduncular cisterns) adjacent to the hypothalamus at the lower midbrain level. (B) The region of interest for the hypothalamus (yellow area) at the level of the upper midbrain. (C) T2-weighted brain magnetic resonance images at the time of diffusion tensor imaging in a representative patient (64-year-old male) show hypothalamic injury (arrows) due to spontaneous subarachnoid hemorrhage.

Supplemental figure 1

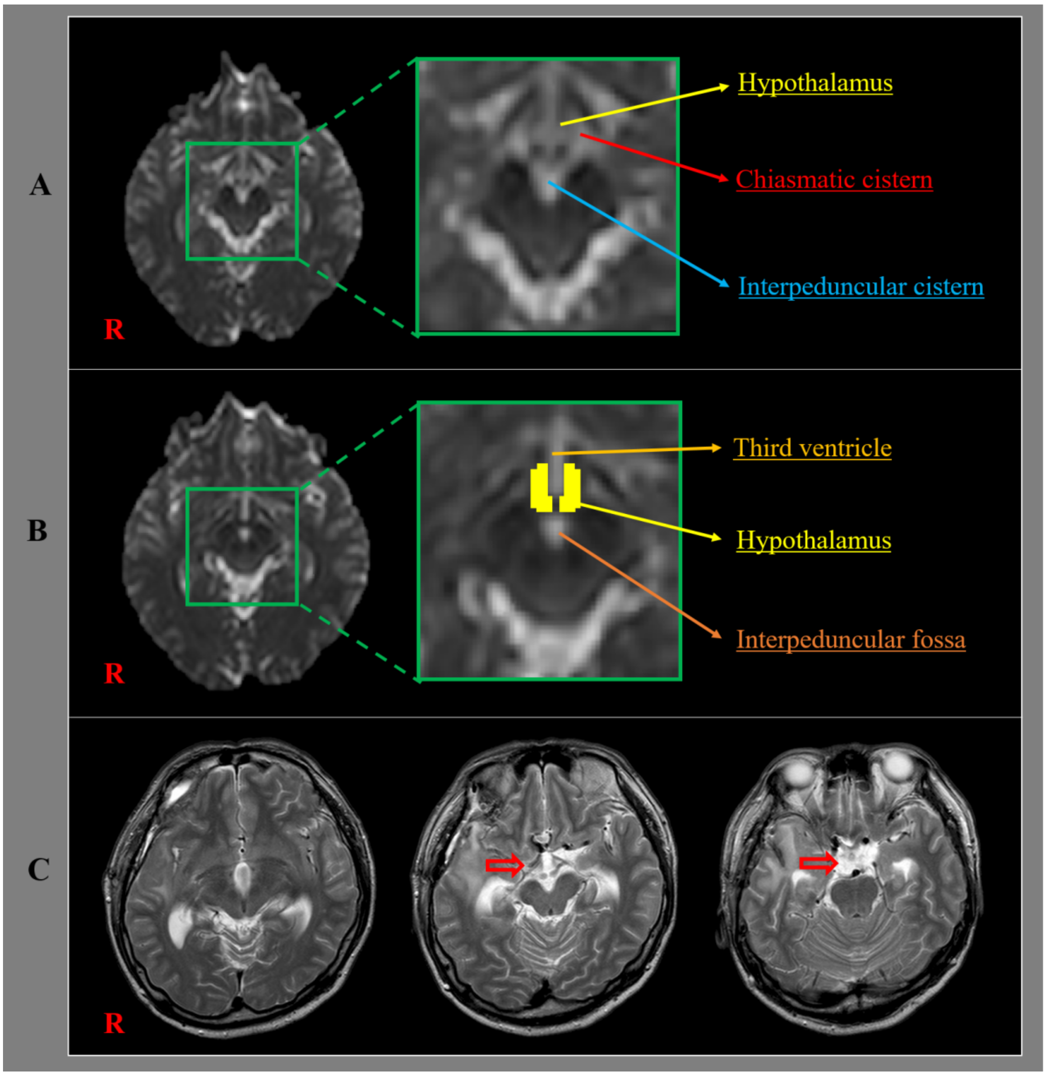

Supplemental table 1. Demographic data for the patient and the control groups.

	Patient group (*n* = 17)	Control group (*n* = 18)
Age (years)	57.65 ± 8.29	51.22 ± 11.73
Range	45 ~ 69	38 ~ 77
Sex, male/female	6 / 11	7 / 11
Duration from spontaneous SAH onset (months)	1.47 ± 1.01	
Modified Fisher grade of SAH (1/2/3/4)	6/0/11/0	
Ruptured artery (ACoA : MCA : PCoA : ICA)	10:5:1:1	

Values represent mean (±standard deviation); SAH: subarachnoid hemorrhage; ACoA: anterior communicating artery; MCA: middle cerebral artery; PCoA: posterior communicating artery, ICA: internal carotid artery

Supplemental table 2. Clinical informations of the patient group.

Patients	BP (systole/diastole)	BT	HR	RR	DOA	Survival
1	150/100	37.3	113	22	5 months	O
2	150/90	36	87	20	16 months	O
3	149/87	37.2	120	22	1 months	O
4	180/100	37.8	110	23	2 months	O
5	181/98	37.8	100	28	3 months	O
6	191/100	36.8	82	20	2 months	O
7	180/110	38.9	112	20	2 months	O
8	190/150	37.2	88	20	2 months	O
9	190/100	36.1	75	22	2 months	O
10	150/80	37.1	75	22	2 months	O
11	160/100	35.2	72	20	5 days	O
12	161/105	35.5	92	18	2 months	O
13	150/90	36.8	92	20	1 months	O
14	170/110	36.6	54	22	3 months	O
15	129/79	36.5	93	24	2 months	O
16	150/80	36	88	24	2 months	O
17	140/90	37.1	61	20	2 months	O

BP: blood pressure, BT: body temperature, HR: heart rate, RR: respiratory rate, DOA: duration of admission

Supplemental table 3. Correlation between clinical information and diffusion tensor imaging parameters in the patient group.

	Systole	Diastole	BT	HR	RR
FA	-0.193	-0.334	0.083	-0.237	0.175
p-value	0.458	0.190	0.750	0.360	0.501
ADC	0.394	0.138	-0.179	0.101	-0.153
p-value	0.118	0.597	0.491	0.701	0.557

FA: fractional anisotropy, ADC: apparent diffusion coefficient, BT: body temperature, HR: heart rate, RR: respiratory rate, *: significant difference between clinical information and the DTI parameters in the patients $p<.05$.
