# Supplementary figures and images for: Hypothalamic injury in spontaneous subarachnoid hemorrhage: a diffusion tensor imaging study
Source: Clin Auton Res. 2020 Nov 28;31(2):321–2. doi: 10.1007/s10286-020-00747-5 (PMC8041696; doi:10.1007/s10286-020-00747-5)

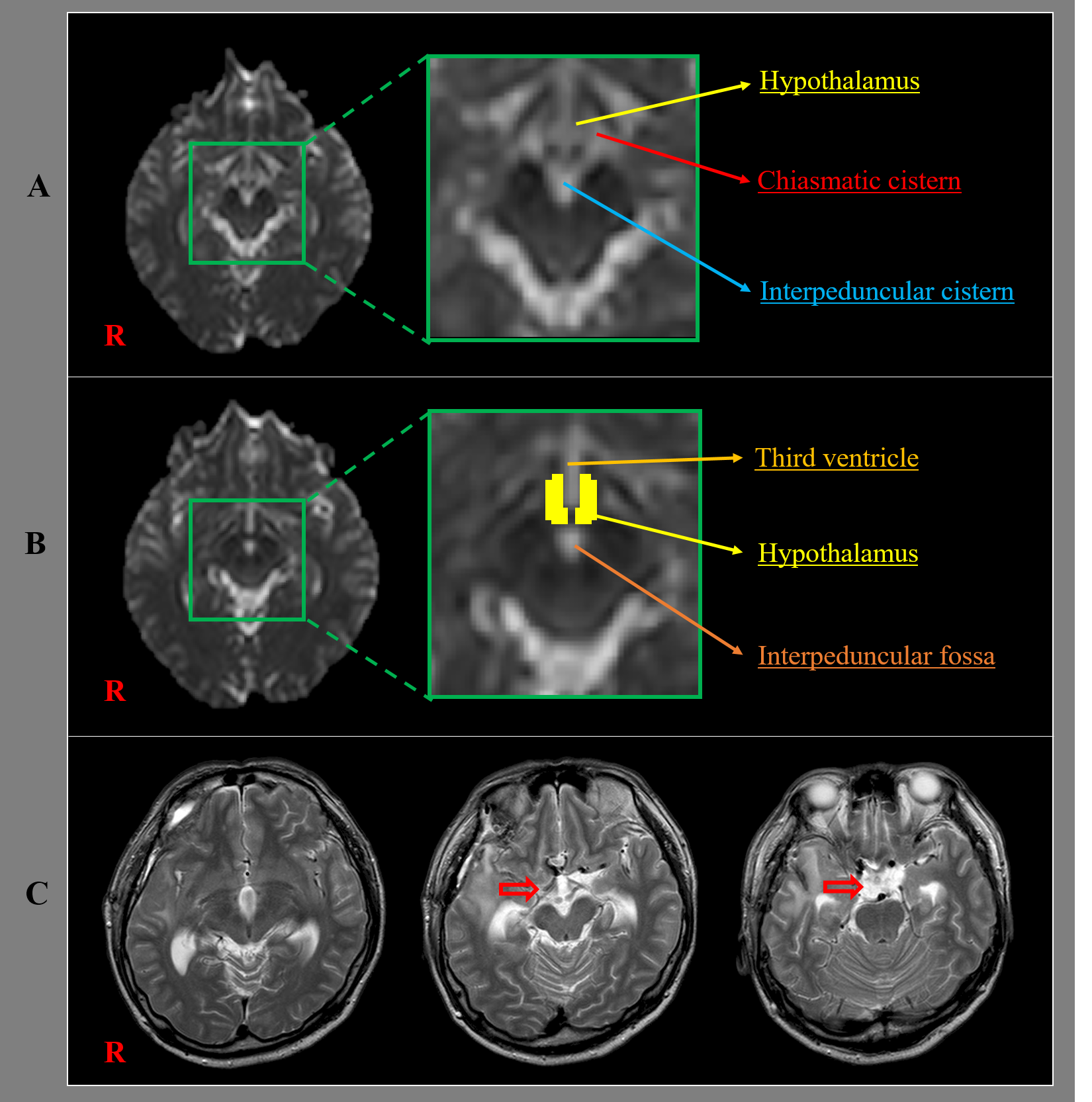

Supplement: Supplementary file 2 — Supplementary file2 (TIF 1,594 kb) [file 10286_2020_747_MOESM2_ESM.tif]
